# K252a Prevents Microglial Activation Induced by Anoxic Stimulation of Carotid Bodies in Rats

**DOI:** 10.3390/toxics11100871

**Published:** 2023-10-20

**Authors:** Ricardo Cuéllar-Pérez, Fernando Jauregui-Huerta, Yaveth Ruvalcaba-Delgadillo, Sergio Montero, Mónica Lemus, Elena Roces de Álvarez-Buylla, Joaquín García-Estrada, Sonia Luquín

**Affiliations:** 1 Microscopía de Alta Resolución, Depto, de Neurociencias, Universidad de Guadalajara, Guadalajara 44340, Mexico; ricardo.cuellarp@academicos.udg.mx (R.C.-P.);; 2 Facultad de Medicina, Universidad de Colima, Colima 28040, Mexico; 3 Centro Universitario de Investigaciones Biomédicas, Universidad de Colima, Colima 28040, Mexico; 4 División de Neurociencias, Centro de Investigación Biomédica de Occidente (CIBO), Instituto Mexicano del Seguro Social, Guadalajara 44340, Mexico

**Keywords:** chemosensitive receptors, cyanide, microglia, BDNF, hypothalamus, amygdala, hippocampus

## Abstract

Inducing carotid body anoxia through the administration of cyanide can result in oxygen deprivation. The lack of oxygen activates cellular responses in specific regions of the central nervous system, including the Nucleus Tractus Solitarius, hypothalamus, hippocampus, and amygdala, which are regulated by afferent pathways from chemosensitive receptors. These receptors are modulated by the brain-derived neurotrophic factor receptor TrkB. Oxygen deprivation can cause neuroinflammation in the brain regions that are activated by the afferent pathways from the chemosensitive carotid body. To investigate how microglia, a type of immune cell in the brain, respond to an anoxic environment resulting from the administration of NaCN, we studied the effects of blocking the TrkB receptor on this cell-type response. Male Wistar rats were anesthetized, and a dose of NaCN was injected into their carotid sinus to induce anoxia. Prior to the anoxic stimulus, the rats were given an intracerebroventricular (icv) infusion of either K252a, a TrkB receptor inhibitor, BDNF, or an artificial cerebrospinal fluid (aCSF). After the anoxic stimulus, the rats were perfused with paraformaldehyde, and their brains were processed for microglia immunohistochemistry. The results indicated that the anoxic stimulation caused an increase in the number of reactive microglial cells in the hypothalamic arcuate, basolateral amygdala, and dentate gyrus of the hippocampus. However, the infusion of the K252a TrkB receptor inhibitor prevented microglial activation in these regions.

## 1. Introduction

Recent studies have found that the carotid body chemoreceptors (CBCs) can modulate the activity of other physiological systems [1]. This modulation can lead, for example, to changes in blood pressure, heart rate, and respiratory rate [2,3]. Moreover, studies have shown that the CBCs are intimately involved in glucose homeostasis and energy metabolism. Specifically, they have been shown to sense changes in glucose levels in the bloodstream and signal to the brain to regulate food intake and energy expenditure. CBCs can also detect the levels of oxygen (O_2_), carbon dioxide (CO_2_), and pH in arterial blood [4,5,6].

Sodium cyanide (NaCN) causes oxidative stress in CBCs by blocking cytochrome C in the mitochondria, inducing a metabolic crisis and activating a chemosensory reflex [7]. This reflex involves the activation of multiple signaling pathways that are initiated by the CBCs and travel through the carotid sinus nerve to the brainstem’s nucleus of the solitary tract (NTS). The NTS is a key site for the integration of sensory information from the CBCs as it receives inputs from various afferent fibers that are involved in the regulation of cardiorespiratory and autonomic function [8,9]. The activation of these pathways is crucial for the survival of the organism, as they trigger a series of responses that increase oxygen delivery to the tissues and maintain cellular homeostasis under hypoxic conditions. From there, the signals are transmitted to other regions of the brain, such as the hypothalamus, hippocampus, and amygdala, where they modulate a wide range of physiological processes, including stress responses, emotional regulation, and memory formation [10,11,12,13].

However, the activation of the chemosensory reflex also leads to an increase in reactive oxygen species (ROS) production and oxidative stress, which can cause cellular damage and trigger an inflammatory response in the brain. Moreover, traces of cyanide can reach the brain and stimulate microglia cells by hypoxia [12]. These responses are mainly mediated by microglia, the resident immune cells of the brain, which become activated, produce proinflammatory cytokines, and transform from a “resting” to an “amoeboid” phase [14,15]. The activation of microglia is a double-edged sword, as it is essential for clearing damaged cells and debris from the brain [16] but can also lead to neuroinflammation and neurodegeneration [17,18]. Moreover, it has been suggested that the activation of microglia and the subsequent inflammatory response may contribute to the development and progression of several neurological diseases, such as Alzheimer’s disease and Parkinson’s disease [19,20]. In fact, studies have shown that the activation of microglia can lead to neuronal damage and death, as well as the production of pro-inflammatory cytokines and reactive oxygen species (ROS) that can damage the surrounding tissue [21,22].

Recent studies have investigated the role of brain-derived neurotrophic factor (BDNF) and its receptor TrkB in the activation of microglia after hypoxic stimulation [23,24]. BDNF is a neurotrophic factor that is involved in the regulation of neuronal survival, differentiation, and synaptic plasticity, and it has been shown to be upregulated in response to hypoxic stimulation [25,26]. It is also a survival and proliferation factor [27,28,29,30] that can modulate the shift between acute and chronic neuroinflammation [31]. It is released by chemo-reflex pathways of the carotid body [32,33], expressed in microglial cells [27,28], and overexpressed in response to nerve damage [34]. TrkB is the high-affinity receptor for BDNF and is expressed in microglia, as well as in neurons and other glial cells [23,35]. The activation of the TrkB receptor in microglia has been shown to promote their proliferation, migration, and phagocytosis, as well as the production of pro-inflammatory cytokines [36,37], and K252a is a potent Trk pan-inhibitor phosphorylator [38,39]. However, it is still unclear how the activation of TrkB in microglia is regulated in response to hypoxic stimulation. 

To further investigate the response of the dentate gyrus of the hippocampus (HDG), arcuate nucleus of the hypothalamus (Arc), and basolateral amygdala (BLA) to anoxic stimuli, we examined microglia transformation after NaCN administration in carotid bodies. We also assessed the role of BDNF and its TrkB receptor as possible mediators of these responses in order to better understand how carotid-body-induced inflammation is regulated. 

## 2. Materials and Methods

### 2.1. Animals and Experimental Design

Male Swiss Wistar rats (N = 12, 280–300 g) were housed in individual transparent polycarbonate boxes with 12:12 h light–dark cycles at a temperature of 22–23 °C with normal laboratory rodent diet and water ad libitum. Until 15 days before the start of the study, they were handled for adaptation and to reduce stress. The rats were grouped, randomly, into the following four groups: (a) *Sham-operated*, received 10 µL/4 min of artificial cerebrospinal fluid (aCSF) within the left lateral ventricle (LV), followed by 100 µL of saline in the isolated carotid sinus (n = 3); (b) aCSF, received 10 µL/4 min of aCSF in the LV four minutes before the injection of NaCN (5 µg/100 g diluted in 100 µL of saline) in the isolated carotid sinus (n = 3); (c) BDNF, received a dose of BDNF (0.25 µg/10 µL of aCSF for 4 min) in the LV four minutes before the injection of NaCN in the isolated carotid sinus (n = 3); and (d) TrkB receptor inhibitor, K252a, received a dose of K252a (25 µg/10 µL of aCSF for 4 min) in the LV four minutes before the injection of NaCN in the isolated carotid sinus (n = 3). Afterwards, the animals were sacrificed by intracardiac perfusion 48 h after the NaCN stimulation; the protocols were performed according to the Guide for the Care and Use of Laboratory Animals of the United States National Institutes of Health.

### 2.2. Substances and Compounds

Substances and compounds used: (a) sodium cyanide (NaCN) (Fluka Biochemika, Sigma, Mexico. Dose: 5 µg/100 g/100 µL saline [5]; icv route, (b) freshly prepared artificial cerebrospinal fluid (aCSF containing: 145 mM NaCl, 2.7 mM KCl, 1.0 mM MgCl, 1.2 mM CaCl_2_, 2.0 mM ascorbate, 2 mM NaH2PO4 (pH 7.3–7.4) [40]; (c) brain-derived neurotrophic factor (BDNF, 0.25 µg/10 µL of aCSF) [41]; (d) TrkB receptor inhibitor, K252a (25 µg/10 µL of aCSF) [42]. The same volume of aCSF was injected to the control rats.

### 2.3. Surgery

The rats were anesthetized with an intraperitoneal (ip) injection of sodium pentobarbital (Pisabental, PiSA Agropecuaria, Hidalgo, Mexico) diluted 1:1 in saline solution (5 mg/100 g); this anesthesia was implemented by an ip infusion of Nembutal in saline (0.063 mg/100 g/min/saline). To ensure deep anesthesia, blink and pinch reflexes were monitored frequently, and the animals were kept artificially ventilated with a tracheal cannula.

### 2.4. Intracerebroventricular Injection in the Left Lateral Ventricle (LV)

Unilateral microinjections of drugs were made using the stellar rat stereotaxic frame (Stoelting Co., Wood Dale, IL, USA). A craniotomy in the left LV was performed with a trephination bur of 1/32 inches to expose the surface of the skull. A glass micropipette (tip diameter 40–50 µm) was inserted using the bregma coordinates: AP = −1.3 mm, L = −1.8 mm, V = 3.6 mm [43]. Drugs were injected through a microinfusion pump (BASI Syringe Pump, MD-1000, West Lafayette, IN, USA) at a rate of 5 µL/min for 2 min, with a connected syringe of a polyethylene tube to the injection cannula. Finally, the cannula was removed, and the craniotomy was sealed with bone wax (Ethicon, Livingstone, UK).

### 2.5. Microglia (Griffonia simplicifolia)

The α-D-galactosil-specific isolectin B4 derived from *Griffonia simplicifolia* seed has shown selective staining in rat microglial cells in both normal and pathologically altered brains [44,45]. To do this, 48 h after icv infusion, rats were transcardially perfused with 37 °C isotonic saline, followed by ice-cold paraformaldehyde ((2.5%) in 0.1 M phosphate-buffered saline (PBS)). After the perfusion, the brains were immediately removed from the skulls and placed overnight in a fixative containing 1% paraformaldehyde in PBS and 30% sucrose at 4 °C. After two days of washing in PBS, the brainstem was sectioned coronally (30 μm) on a vibratome (Leica VT-1000E). Brain sections were incubated in a solution containing PBS 0.1M/Triton 0.1%/Albumin 0.1% (PBS-T-A) for 15 min at room temperature then transferred to a cation solution (PBS 0.1M, MgCl_2_, MnCl_2_, CaCl_2_) for 10 min. After that, the tissues were washed with PBS and left to incubate in free-floating with Griffonia *Simplicifolia* Lectin I (GSL I) non-conjugated goat isolectin B4 (1:100, Vector L1104, Burlingame, CA, USA) for 13 h at 4 °C. Then, the tissues were washed and placed in a Tris-buffered saline solution, 0.1 M (TBS), with Triton X-100 0.1% (TBS-T). To block non-specific reactivity in the tissues, they were incubated in TBS-T with 2% normal rabbit serum for 60 min at room temperature. After washing with PBS-T-A, the brain slices were incubated with the primary antibody, non-conjugated anti-griffonia (Bandeiraea) simplicifolia lectin (GSL, BSL) from goat, at a dilution of 1:100, Vector AS-2104, Burlingame, CA, USA, for 15 h at 4 °C. Then, the slices were washed with PBS-T-A and incubated with the secondary antibody for 2 h at room temperature. Subsequently, the slices were incubated with the ABC complex (Vectastain kit, Vector Labs, Burlingame, CA, USA) for 2 h at room temperature, washed with PBS-T-A, followed by a wash with PBS, and a final wash with Tris-HCl 0.1 M. The peroxidase reaction product was visualized by incubating the slices for 8 min with a chromogenic solution to obtain a brown color (3,3′-diaminobencidina 0.07% and H_2_O_2_ 0.01%) (Aldrich, St. Louis, MO, USA). Then, the slices were washed with PBS, 0.1M; this procedure is summarized in Table 1. Finally, the slices were mounted on slides, dried and dehydrated, and covered with Entellan (Merck, Darmstadt, Germany). The slices were analyzed with a Leica microscope (DMi8, Leica Microsystem, Wetzlar, Germany), and the images were recorded with the LAS AF Lite program (v4.6, Leica, Microsystem, Wetzlar, Germany). Morphometric analysis was performed by an investigator who was unaware of the identity of the experimental groups. For quantitative investigation, 10 equidistant pairs of adjacent sections were used. The numerical density of microglia cells was estimated by using the optical disector method in the brain regions mentioned above, using the total thickness of the slices for a disector height of 20X [46], and a two-dimensional point intersect template was calibrated so that the area associated with each point was known (125 × 75 μm). Microglia cells for which the clearest profiles fell within the disector volume and did not touch the left and bottom borders of the superimposed counting template nor the superior were counted. The estimated volume was indirectly calculated by multiplying the template × slice thickness × number of analyzed slices by the numerical density value using the disector method; this gives us an indirect estimation of the total number of microglia cells in each analyzed region [47]. In addition, the percentage of microglia cells with different morphologies was also evaluated. The cells were classified into five morphological types: Type I, cells with few cellular processes (two or less); Type II, cells displaying three to five extensions; Type III, cells with numerous (more than five) and long extensions and with a small cell body; Type IV, cells with large somas and thick and retracted extensions; and Type V, ameboid-shaped cells with numerous short extensions. For each animal, 120 cells were analyzed in each region ipsilateral to the CBC stimulation [48].

### 2.6. Statistical Analysis

Values are presented as means ± SEM, and the number and morphology of microglia cells were compared among the four conditions (aCSF, BDNF, K252a, and *Sham-operated*) using a one-way ANOVA followed by Tukey’s post hoc test. Values of *p* < 0.05 were considered statistically significant. The statistical analysis was performed using GraphPad Prism 5 (GraphPad Software Inc., San Diego, CA, USA).

## 3. Results

### 3.1. Stimulation of the CBC with NaCN Increases the Density of Microglia in the Amygdala, Dentate Gyrus Hilus, and Arcuate

**Figure 1:**
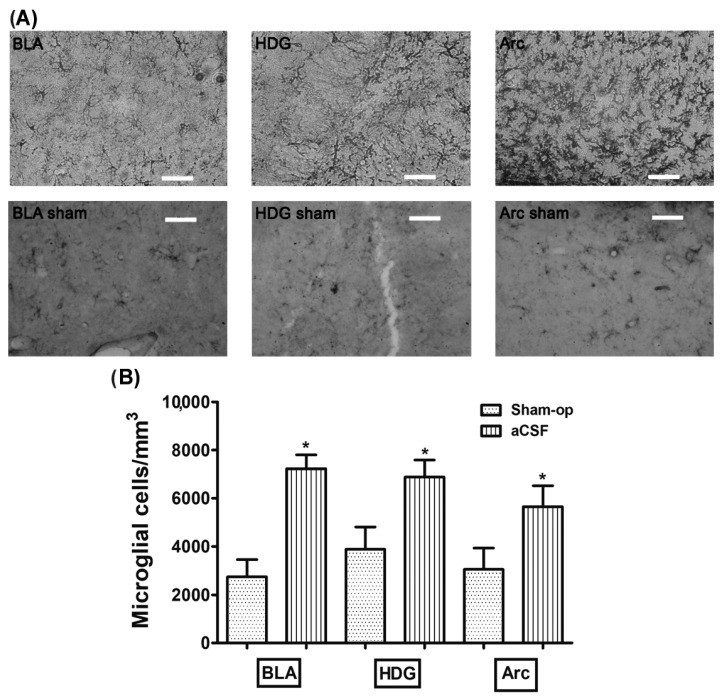
Stimulation of CBCs with NaCN increases the density of microglia in brain regions involved in the carotid chemoreflex. (**A**) The upper panel shows glial reactivity in the basolateral amygdala (BLA), the hilus of the dentate gyrus (HDG), and the arcuate nucleus (Arc), produced by aCSF infusion into the left LV prior to stimulation of CBCs with NaCN. (**B**) Shows the density of microglia cells in the analyzed regions. These values are presented as means ± S.E.M. The asterisk sign (*) indicates a significant difference with *Sham-operated* group (ANOVA, Tukey’s post hoc test). Scale—50 µm.

The rats infused with aCSF prior to CBC stimulation with cyanide had an increase density of microglial cells in the basolateral amygdala (BLA), dentate gyrus hilus (HDG), and arcuate (Arc) (7229 ± 574, 6882 ± 706, 5647 ± 874 microglia cells/mm^3^, respectively) compared to the reactivity displayed in the BLA, HDG, and Arc in the *Sham-operated* group (2743 ± 711, 3879 ± 927, 3048 ± 880 microglia cells/mm^3^, respectively; *p* = 0.0007, *p* = 0.027 and *p* = 0.048, respectively) (Table 2, Figure 1A,B). Additionally, we found that the microglial morphology observed in the aCSF + NaCN group in greater quantity was type II in BLA (58%), type III in HDG (36%), and type II in Arc (54%), while the *Sham-operated* group mostly presented type I cells in the three regions analyzed (BLA 89%, HDG 57%, and Arc 57%) in Table 3. These results indicate that anoxic stimulation of the CBC with NaCN activates the immune response in brain regions involved in the chemosensory response.

### 3.2. The Infusion of K252a in the Left LV after the Injection of NaCN in the CBC Prevented Microglial Reactivity in the Basolateral Amygdala

**Figure 2:**
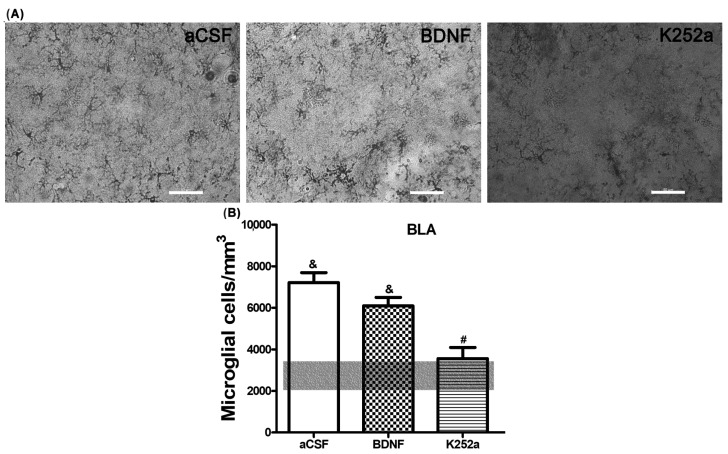
The K252a inhibitor does not increase microglial reactivity in the basolateral amygdala. (**A**) Decreased number density and morphological complexity were observed by K252a infusion into the left LV after NaCN injection into the CBC. (**B**) The transparent bar corresponds to the number of microglia cells in the *Sham-operated* group (2743 ± 711) that are statistically different to the aCSF and BDNF groups. These values are presented as means ± S.E.M. The ampersand sign (&) indicates a significant difference with the *Sham-operated* group, and the number sign (#) indicates a significant difference with the BDNF-infused group (ANOVA, Tukey’s post hoc test). Scale—50 µm.

**Figure 3:**
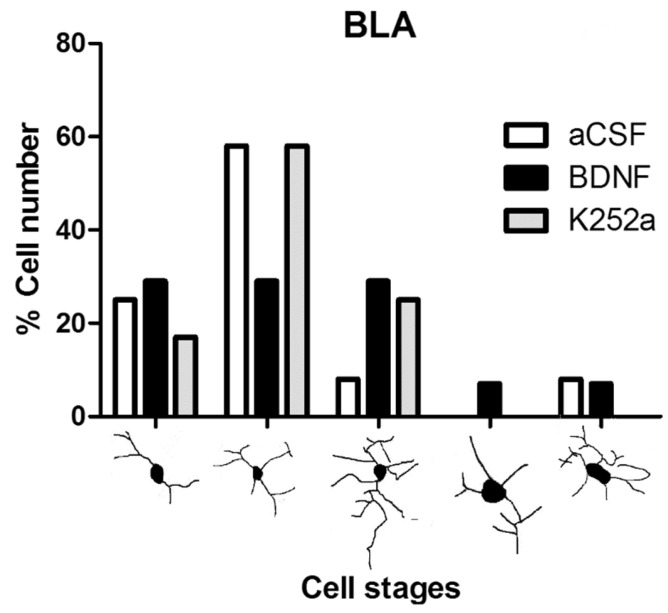
Morphological analysis of microglia cells in the basolateral amygdala. The graph shows the proportion of each morphological type (I–V) in the groups infused with aCSF, BDNF, or K252a. The main form observed was II, corresponding to the quiescence stage in the aCSF and K252a groups, and BDNF is probably a modulator of the activation state.

When the microglial reactivity in the basolateral amygdala (BLA) was analyzed, it was found that the density of microglia cells increased in the aCSF + NaCN group (7209 ± 488 microglia cells/mm^3^) and in the BDNF + NaCN group (6095 ± 408 microglia cells/mm^3^) compared to the *Sham-operated* group (*p* = 0.0005 and *p* = 0.0009). On the other hand, when the inhibitor K252a was administered, no reactions to the anoxic stimulus were observed (3556 ± 536 microglia cells/mm^3^), resulting in a similar number to the *Sham-operated* group (2743 ± 711 microglia cells/mm^3^ *p* = 0.459) (Table 2, Figure 2A,B). In relation to the morphological analysis, a diversity of microglial cells was present in this region. The K252a + NaCN group showed predominant type II cells (58%). The rats that received the icv infusion of BDNF + NaCN showed type I cells (a few cytoplasmic arborizations), type II cells (showing three to five branches), and type III cells, with small soma and numerous and long cytoplasmic branches (29% for each type) (Table 3, Figure 3).

### 3.3. Infusion of BDNF or K252a in the Left LV Prior to the CBC Stimulation Prevented the Increase in Microglial Cell Density in the Hilus of the Dentate Gyrus

**Figure 4:**
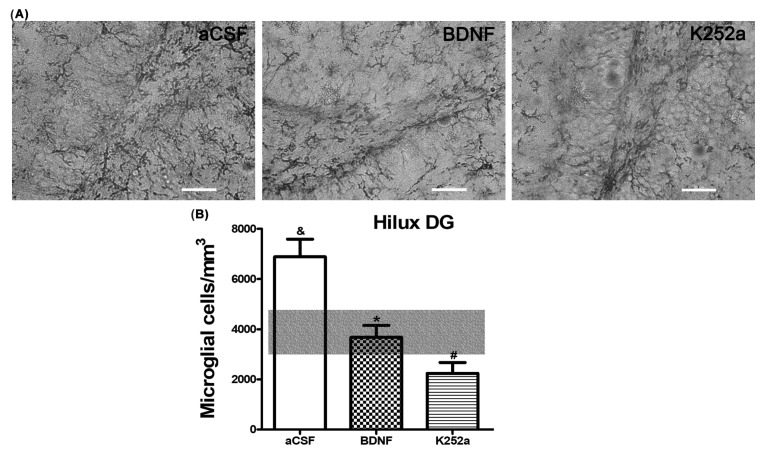
BDNF or K252a does not increase microglial cell density in the hilus of the dentate gyrus of the hippocampus. (**A**) BDNF or K252a infusion into the left LV prior to stimulation of CBCs with NaC has a down-regulating effect on active microglial cells. (**B**) The transparent bar corresponds to the number of microglia cells in the *Sham-operated* group (3879 ± 927) that are statistically different to the aCSF group. These values are presented as means ± S.E.M. The ampersand sign (&) indicates a significant difference with the *Sham-operated* group, the asterisk sign (*) indicates a significant difference with aCSF, and the number sign (#) indicates a significant difference with BDNF (ANOVA, post hoc Tukey test). Scale—50 µm.

**Figure 5:**
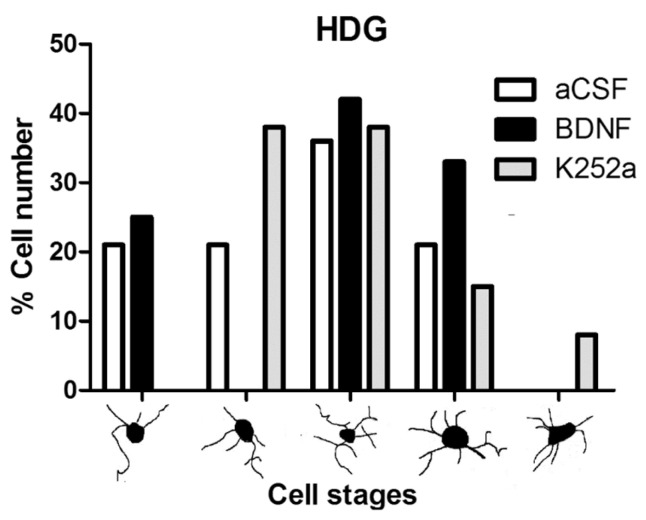
Morphological analysis of microglia cells in the hilus of the dentate gyrus. The graph shows the proportion of each morphological type in the groups infused with aCSF, BDNF, or K252a. Although some forms correspond to phagocytic microglia acting as intracerebral macrophages, these numbers are due to the normal variability of resident, nonproliferative microglia.

In the case of the dentate gyrus of the hippocampus, we found that the rats infused with BDNF + NaCN or K252a + NaCN had less microglial reactivity (3694 ± 491 microglia cells/mm^3^ and 2241 ± 433 microglia cells/mm^3^) compared to the aCSF + NaCN group (*p* = 0.0004 and *p* = 0.0000003, respectively) and that their numbers were similar to the *Sham-operated* group (*p* = 0.855 and *p* = 0.083, respectively) (Table 2, Figure 4A,B). There were no significant differences between these groups in relation to the microglia population. In this area, the microglial morphology in the BDNF group corresponded to type III (42%). The group infused with the inhibitor K252a exhibited types II and III (38% both) (Table 3, Figure 5).

### 3.4. Blocking the TrkB Receptor Prior to CBC Stimulation Prevented the Increase in Microglia Density in the Arcuate Nucleus of the Hypothalamus

**Figure 6:**
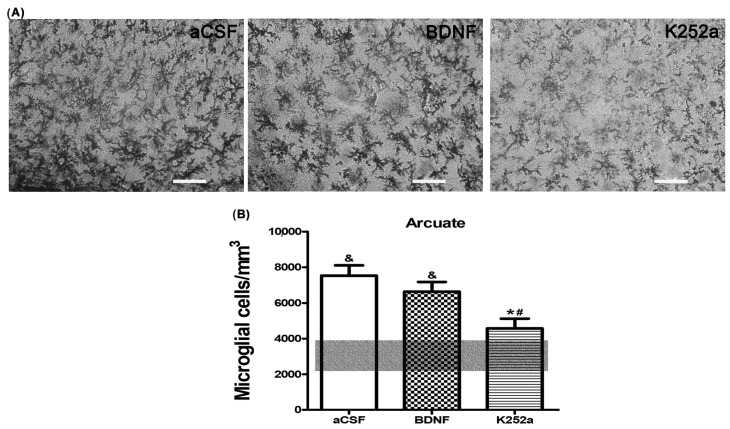
TrkB receptor blockade does not increase the density of microglia in the arcuate nucleus of the hypothalamus. (**A**) K252a infusion showed microglial reactivity (3288 ± 450 microglia cells/mm^3^) similar to the *Sham-operated* group (*p* = 0.811), but the decrease was not significant compared to BDNF infusion. (**B**) The transparent bar corresponds to the number of microglia cells in the *Sham-operated* group (3048 ± 880) that are statistically different to the aCSF and BDNF groups. These values are presented as means ± S.E.M. The ampersand sign (&) indicates a significant difference with the *Sham-operated* group, the asterisk sign (*) indicates a significant difference with aCSF and the number sign (#) indicates a significant difference with BDNF (ANOVA, Tukey’s post hoc test). Scale—50 µm.

**Figure 7:**
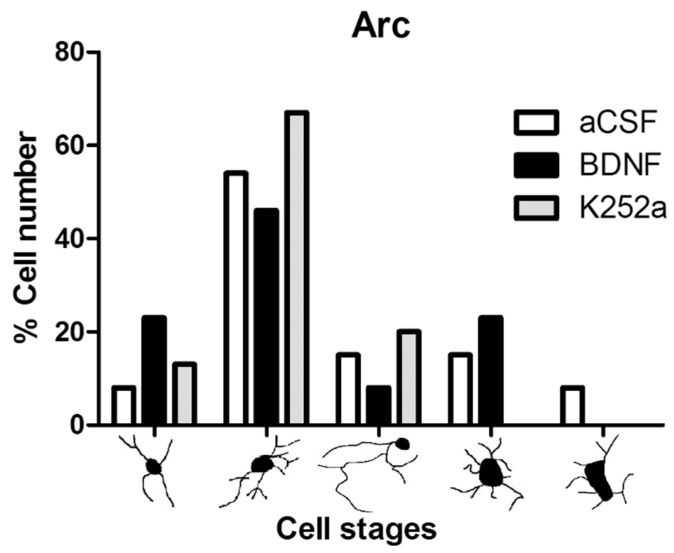
Morphological analysis of microglia cells in the arcuate nucleus of the hypothalamus. The graph shows the proportion of each morphological type in the groups infused with aCSF, BDNF, and K252a. The highest proportion found in the arcuate nucleus presents type II, which corresponds to a resting phenotype; stimulation of CBCs with NaCN was not sufficient to achieve microglial activation.

Finally, upon analyzing the arcuate nucleus of the hypothalamus, we found that the rats infused with BDNF 4 min before the injection of NaCN had a similar amount of microglial cells (5012 ± 621 microglia cells/mm^3^ *p* = 0.774) to the group infused with aCSF. On the other hand, the group of rats infused with the Trk inhibitor for the BDNF receptor, K252a, showed microglial reactivity (3288 ± 450 microglia cells/mm^3^) similar to the *Sham-operated* group (*p* = 0.811) (Table 2, Figure 6A,B). The predominant microglia morphology in the three different NaCN treatments consisted of small cellular bodies with three to five cytoplasmic processes, corresponding to type II cells (46% BDNF + NaCN; 67% K252a + NaCN) (Table 3, Figure 7).

## 4. Discussion

In this study, we first evidenced microglial changes in regions related to the reflex chemosensory response after exposure to sodium cyanide (NaCN). Subsequently, we investigated the role of brain-derived neurotrophic factor (BDNF) and its receptor tyrosine kinase B (TrkB) in microglial activity induced by NaCN stimulation. Our results demonstrated that the manipulation of the BDNF–TrkB system was able to suppress the inflammatory response associated with microglia in the hypothalamus, hippocampus, and amygdala.

We briefly stimulated the carotid body with a sodium cyanide solution to induce a localized hypoxic response [5]. Then, we examined the potential relationship between NaCN stimulation and microglia response, which may involve BDNF [33,49,50]. To this end, one group of animals received an icv infusion of aCSF, while another group was administered BDNF, and a similar group was subjected to the TrkB inhibitor [51,52,53]. We compared the results to a control group of rats. The animals were sacrificed 48 h after the NaCN stimulus to ensure microglial reactivity. This model has been previously proven to induce cyanide toxicity by binding to the mitochondria, blocking electron transport, and inhibiting respiration [54], so it can cause severe CNS depression and death [55]. Our group has previously used this model to analyze c-fos activity and GluR2/3 receptor expression [56,57]. Since our previous studies evidenced that activity changes were not restricted to neurons, we proposed that astrocytes and microglia should be involved in the cerebrum’s response to NaCN CBC stimulation. Furthermore, previous research validated our model, evidencing that after icv infusion, exogenous BDNF is dispersed throughout areas near the ventricles. Its diffusion depends on the distribution of its receptor TrkB [58,59]. However, incomplete forms of TrkB without the kinase domain may limit its diffusion [60].

Regional susceptibility was evaluated through measuring microglial reactivity in regions such as the hippocampus, amygdala, and hypothalamus. The hippocampus is an area related to memory and spatial learning that is particularly affected in animals exposed to intermittent hypoxia [61,62,63] or in patients with obstructive sleep apnea [64]. It has been demonstrated that hypoxic events may induce a loss of neurons in the hippocampus [65,66]. In addition, studies on ischemia–reperfusion evidenced that all layers of the dentate gyrus showed an activated form of microglia and greater cell numbers compared to a *Sham-operated* group [67]. Evidence also exists showing that the BDNF synthesized by microglia is present in various regions of the CNS during the course of various neurological disorders such as traumatic injury [68] and ischemia [69]. In our study, aCSF caused an increase in the density of microglia in the hilus region of the dentate gyrus of the hippocampus compared to the *Sham-operated* group, while exogenous BDNF and the inhibitor K252a caused a decrease in microglial reactivity after the NaCN-induced chemotoxic hypoxia. These effects were not observed in the other regions already analyzed. This was probably due to the fact that the dentate gyrus is the least excitable region since it has a strong inhibitory GABAergic innervation with very few excitatory synapses [70]. BDNF released by microglia acts on different types of neighboring cells, such as neurons, astrocytes, and possibly the same microglia, through a paracrine pathway. Yet, our results revealed that the regional reactivity of microglia in the dentate gyrus was lower compared to the other studied regions. In this line of evidence, other experiments demonstrated that microglial reactions may occur sooner in the dentate gyrus than in the CA1 zone, at least under hypothermic ischemia [71]. Then, it seems that this region is more resistant to an ischemic/hipoxic injury, while the CA1 region could be more vulnerable [72,73]. Therefore, this suggests that the effects of cyanide stimulation and BDNF/TrkB must vary according to regional parameters. 

The basolateral amygdala (BLA) is also a region involved in the chemosensory reflex pathway that receives stimuli through the NTS from the brainstem [74]. We noted here that groups infused with aCSF and BDNF showed a greater number of microglia cells compared to the *Sham-operated* group, while tissue from the rats that received the inhibitor K252a showed a low expression of microglia compared to the same groups. Again, blocking the TrkB receptor in the BLA decreased the number of microglia after the hypoxic stimulation. This effect suggests that the stimulating action of BDNF on microglial reactivity caused by the hypoxic stimulus was inhibited. The rats that received the inhibitor K252a or aCSF showed microglia morphology type II (resting), while in the group with infusion of BDNF, the three predominant forms were observed: I, II, and III (activated). In other experiments, microglia have been detected 14 days after a cardiac infarction; these microglial cells show hypertrophy of the cytoplasm and their processes are retracted, while the *Sham-operated* group had small and thin cytoplasmic processes, indicating that the microglia were in their resting form [75]. An elevated synthesis of BDNF has been confirmed in different regions of the CNS after an ischemic event [76]. With this, the results of our work could indicate that the neurotrophin BDNF promotes the transformation of microglia acting in cells that have a different amount of TrkB receptors, so the effects can differentially be detected in these cells. Since the BLA region is an important area for emotional and cognitive processing, it follows that stimulation with NaCN could cause cognitive function disorders. More studies are needed to evaluate the effects of this stimulation on cognition and emotion. 

Finally, we studied the regional susceptibility of the arcuate nucleus of the hypothalamus to NaCN stimulation since the chemo-reflex response to hypoxia is coordinated by a brain stem pathway, which includes the solitary tract nucleus (NTS), the rostral ventrolateral medulla (RVLM), and the medullary pathways projecting to the paraventricular nucleus of the hypothalamus [77]. The arcuate nucleus, which has abundant TrkB receptors, also regulates behavior, sleep, energy expenditure, and feeding [78,79]. We evidenced arcuate nucleus susceptibility to NaCN stimulation since more microglial cells were counted in the groups with aCSF and BDNF infusions compared to the *Sham-operated* group. However, we also evidenced that blocking the TrkB receptor with the inhibitor K252a prevented the increase in microglia, making the number of microglia similar to the amount in the *Sham-operated* group. As stated before, microglia have a primary function of responding to changes in the extracellular environment, protecting neurons, and promoting the repair of nervous tissue in pathological situations [21,80,81]. When faced with damaging signals, microglia change morphologically and functionally, resulting in migration, proliferation, and increased production and secretion of proinflammatory cytokines and neurotrophic factors. Such changes are known as “microglial activation” or microgliosis [82,83,84,85,86,87]. The activated microglia can modulate the inflammatory response to produce mediators that work to eliminate the source of the inflammatory stimulus. However, an excessive activation of microglia, as can occur in hypoxia, may result in chronic neuroinflammation and promote neurodegenerative diseases. Then, our findings suggest that the BDNF–TrkB system may play a critical role in regulating microglial activity and protecting against neurodegeneration. Studies from other groups shed light on the potential therapeutic benefits of targeting microglial activity in response to hypoxic stimuli. One study found that melatonin may be a viable pharmacological target for protecting against hypoxic-derived neuronal damage, as it attenuated the toxicity of chemical hypoxia on microglia through the involvement of SIRT1 and AMPK [87]. Another study demonstrated that therapeutic hypothermia could be effective in attenuating microglial activation and migration following brain damage caused by an ischemic stroke or a traumatic brain injury [88]. In addition, another study evidenced that microglia have the ability to secrete constitutive BDNF in a non-stimulated phase, which has anti-inflammatory effects that inhibit microglia activation [89,90]. Together, these findings suggest that targeting microglial activity may be a promising approach for treating neuroinflammatory conditions and protecting against hypoxic-induced neuronal damage. However, further research is needed to determine the optimal time window for therapeutic interventions and to better understand the mechanisms underlying these effects. 

## 5. Conclusions

We demonstrated that the stimulation of CBCs with NaCN induces a strong inflammatory response mediated by microglial cells in the arcuate nucleus, basolateral amygdala, and dentate gyrus hilus of the hippocampus. Furthermore, we evidenced that brain-derived neurotrophic factor (BDNF) and its receptor TrkB are involved in NaCN stimulation. The TrkB receptor inhibitor, K252a, significantly inhibited microglial reactivity, indicating a role for the tyrosine-kinase receptor in these changes. Thus, TrkB receptor inhibition could diminished chronic neuroinflammation after anoxic stimulation.

## Figures and Tables

**Table 1:** Summarizes the general procedure of the immunohistochemical process.

(1) Chemical Union of GRIFFONIA to Glycocalix.	(2) Incubation with Primary Antibody (Ab).	(3) Incubation with Secondary Ab.	(4) Incubation with Avidin–Biotin Complex (ABC)
*Griffonia simplicifolia* Lectin I non-conjugated goat isolectin B4 1:100	Anti-griffonia (Bandeiraea) simplicifolia lectin goat 1:100	Biotinylated rabbit anti-goat IgG 1:150	Sandwich-type reaction and color development in chromogenic solution

**Table 2:** Multiple comparisons on the number of microglia cells in response to each treatment.

	Area		
Drug	BLA	HDG	Arc
aCSF	7209 ± 488 *	6882 ± 706 *	5647 ± 873 *
BDNF	6095 ± 408 *	3694 ± 491 ^#^	5012 ± 621
K252a	3556 ± 536	2241 ± 433 ^#^	3288 ± 450 ^#^
*Sham-operated*	2743 ± 711	3879 ± 927	3048 ± 880 *

These values are presented as means ± M.E.S. The asterisk sign (*) indicates a significant difference with the *Sham-operated* group, and the number sign (#) indicates a significant difference with the aCSF group (ANOVA, post hoc Tukey test).

**Table 3:** Morphology of microglia type in each area after NaCN infusion in CBCs for each treatment.

	Area		
Drug	BLA	HDG	Arc
aCSF	II (58%)	III (36%)	II (54%)
BDNF	I, II, III (29% each)	III (42%)	II (46%)
K252a	II (58%)	II, III (38% each)	II (67%)
*Sham-operated*	I (89%)	I (57%)	I (57%)

## Data Availability

The data presented in this study are available on request from the corresponding author.
